# Architecture Design and VLSI Implementation of 3D Hand Gesture Recognition System †

**DOI:** 10.3390/s21206724

**Published:** 2021-10-10

**Authors:** Tsung-Han Tsai, Yih-Ru Tsai

**Affiliations:** Department of Electrical Engineering, National Central University, Taoyuan City 32001, Taiwan; raymail11@dsp.ee.ncu.edu.tw

**Keywords:** VLSI, ASIC, FPGA, hand gesture recognition, SAD matching, object labeling

## Abstract

With advancements in technology, more and more research is being focused on enhancing daily life quality and convenience. Along with the increase in the development of gesture control systems, many controllers, such as the keyboard, mouse, and other devices, have been replaced with remote control products, which are gradually becoming more intuitive for users. However, vision-based hand gesture recognition systems still have many problems to overcome. Most hand detection methods adopt a skin filter or motion filter for pre-processing. However, in a noisy environment, it is not easy to correctly extract interesting objects. In this paper, a VLSI design with dual-cameras has been proposed to construct a depth map with a stereo matching algorithm and recognize hand gestures. The proposed system adopts an adaptive depth filter to separate interesting foreground objects from the background. We also propose dynamic gesture recognition using depth and coordinate information. The system can perform static and dynamic gesture recognition. The ASIC design is implemented in TSMC 90 nm with about 47.3 K gate counts, and 27.8 mW of power consumption. The average accuracy of each gesture recognition is 83.98%.

## 1. Introduction

In this explosion of the digital information era, computers and many appliances play important roles in our lives to make them more convenient. As technology is advancing, more and more research is being focused on gesture recognition. Gesture recognition helps us to connect with deaf and people who cannot speak using sign language, control robots, and home appliances without a controller. In human–computer interfaces (HCI), gesture recognition is an important topic in which breakthrough work needs to be achieved [1]. Traditional gesture recognition devices are not convenient and are constrained by the environment, such as needing data gloves [2]. As a result, a great deal of research has been recently focused on vision-based gesture recognition [3]. Through sensors, hand gesture recognition algorithms can provide a more intuitive and convenient way for users to learn a device for a specific application. Other than helping users to control devices without a physical interface, it will save users from feeling irritated through the new intuitive control technology. The products with have cheap prices, higher stability, and small sizes, which will help to achieve more adaptability from users. Gesture recognition is an important topic that has a high potential value in HCI academic research.

Gesture recognition has a wide range of applications, such as:

- Helping the hearing impaired;
- Recognizing sign language;
- Helping mobile drivers to control some devices without looking at them;
- Improving public health (by eliminating the need to touch public devices);
- Manipulating in virtual environments.

The research on hand gesture recognition based on vision is always categorized into two groups. The first uses a single image sensor to capture video. In this category of research, an interesting part, i.e., the hand, is separated using a clean background [4] or motion information [5]; however, it is not an efficient method for developing a real-world product, because it is difficult for users to perform gestures when there is only a clean background or without moving the face. However, in the case of using a single CMOS camera, the traditional gesture recognition algorithm has a very poor recognition rate in complex scenes, and hand gestures can only be recognized in a simple scene.

The other category of gesture recognition methods uses an infrared camera, such as Kinect or a dual-camera like the ZED Camera [6], to obtain the depth information. When developing a dual-camera hardware architecture system, a depth map is constructed to acquire more information about the environment. It can deal with the environment when there is no clean background, or even in some complex environments. With this concept, many products have been developed and are on the market and are being used in many types of research so far, such as Kinect and Real Sense. Both of these products use an infrared camera to construct a depth map and develop different information. However, these products are always have a relative size from the user’s view and are too expensive. For all environments, especially in outdoor scenes, depth-sensing devices may not be suitable at all times of the day.

As CMOS technology develops rapidly, single- or dual-camera can be made as small and as cheaply as possible. As high integration on the system-level design trend, the CMOS-based VLSI design is easy for designers to make a system-on-a-chip. The dual-camera architecture system proves that it can reach the level of efficiency of other products and reduce system costs. In addition to being used to control appliances, it can also be used in a virtual environment; however, a dual-camera system is dominated by the vision-based algorithm. Additionally, to design hardware architecture with a dual-camera system, not only is the system quality an issue, but the computation power is as well.

To let users feel unrestrained and comfortable when using the product, a hardware architecture system is developed using a dual camera. In [7] and [8], skin color detection and multi-scale color detection were used to find a target. However, the high complexity of the algorithm and the large computation burden were drawbacks. It will consume extensive resources in hardware implementation. In our previous work [9,10], an implementation of FPGA for a 3D hand gesture architecture system under a complicated environment is provided.

In this paper, a hardware architecture system is proposed to improve the execution speed while maintaining a high efficiency. It can recognize one static gesture as “fist”, five dynamic gestures as “up, down, left, right” and 3D gestures as “push”, respectively. The two dynamic gestures can interact with each other, so the system can recognize gestures like “push up”, “push down”, etc. On the other hand, the design is implemented in the SMIMS development board using Xilinx Artix-7 to demonstrate the HCI system.

The remainder of the paper is organized as follows. Section 2 includes some related works on depth extraction, the area of interest detection, and hardware designs. In Section 3, details of the proposed architecture system are discussed. In Section 4, the experimental results and specifications of the proposed architecture system are shown. The conclusions are provided in the last part of the paper. Overall, the contributions of this paper are listed as:

- We use a lower-priced dual-camera device to construct a depth map and achieve real-time 3D hand recognition;
- We implement the whole system in VLSI design and demonstrate the HCI system to verify the overall architecture.

## 2. Related Works

As vision-based hand gesture recognition has become a hot research topic, more and more products and algorithms are being proposed targeting it. In the field of gesture recognition research, hand segmentation is an important element. The completeness of segmentation and the removal of noise will seriously affect the difficulty of recognition. The most common method is to use a skin color detector to filter the background. Color filters are usually used to detect areas of skin, and some research has added depth information to acquire more information about the environment.

### 2.1. Depth Information Extraction

There are several devices that can be used to construct the depth map; these devices fall into two categories. The first one is infrared cameras, such as Kinect and RealSense. Kinect is used in many types of research due to the high quality of the image and its speed efficiency. However, the price is high and the device is large, so it is difficult to set it up in real-world applications. The second category of devices includes dual-camera systems. These construct a depth map using the same concept as human eyes. Two sensors are used to calculate the same object’s coordinates to acquire the disparity and then the disparity is transformed into a depth map.

The algorithm of environment construction through a dual-camera system can be classified into two methods. The first is a dense disparity map, which has two classifications. The second method is the global method algorithm, which has a high accuracy but at the cost of a high computing time. Therefore, these methods are difficult to implement in embedded systems. The global method contains belief propagation [11], scanline optimization [12], and dynamic programming [13]. The disadvantage of the local method algorithm is that it is a block-based matching algorithm, so it will sometimes cause matching errors and will result in a reduction in accuracy for the depth map. The common methods to calculate sparse disparity map include SAD (sum of absolute differences), graph cut [14], SSD (sum of squared differences), etc. These methods are used to perform edge and corner detection. A sparse disparity map has a low complexity, but the efficiency of the depth map is not good.

### 2.2. Area of Interest Detection

In a gesture recognition system, the area of interest detection is the key area that needs a good gesture recognition rate with a high accuracy. The simplest way to achieve area of interest detection is through background subtraction [15]. Mesbahi et al. [16] proposed a gesture recognition method using background subtraction and convexity defects. First, background subtraction is used to delete useless information, then the contour segmentation of the hand image needs to be determined, and the contour image to calculate convex hulls and convex defects is then calculated. It also uses feature analysis and identification parameter extraction for classification and recognition. Through comparison of the area of interest with the background model, the accuracy of the segment in the foreground object becomes high. However, the disadvantage of such a method is that the system needs to save the information of the background model and the foreground cannot contain other moving objects. It is difficult to use this method in a complex environment.

Another method uses skin-like color detection and motion information to segment the area of interest. Hasan et al. [17] used two different methods to segment the input hand image; subdivision of skin color using an HSV color model and thresholding technology to capture the shape of hands and their features. An improved direction analysis algorithm was used to determine the relationship between statistical parameters from the data, and through hand gesture direction to calculate the slope and trend of the hand. It is a good way to detect the hand part and the complexity is low in this case. However, this method cannot be used in environment where too many objects have skin-like colors.

Haar-like feature with Adaboost is a robust method to segment the hand part. A two-level real-time hand gesture recognition has been introduced, combining Haar-like features to implement pose detection and the AdaBoost algorithm was based on stochastic context-free grammar [18]. Machine learning is being used in many systems at the moment; however, due to the high computational resource demands, it is difficult to develop hardware gesture recognition work in an embedded system. With the depth map of dual-camera and Kinect, however, researchers can use the information to segment the hand part as the nearest object [19,20,21]. This is an efficient way to find an area of interest, even when the scenario is in a complex environment, which is one of the reasons why the proposed system adopts a stereo matching module.

### 2.3. Hardware Design

There are some related works in which the stereo vision algorithm is used in gesture recognition. Raj et al. [22] used skin color detection to perform hand segmentation and convert the image into a binary signal and find the centroid of a hand. The hand recognition is done by counting the number of zero-to-one (black-to-white) transitions from left to right to determine the number of fingers. Because the number of fingers can be identified by spreading the fingers, as long as the number of fingers is the same, different gestures may be seen as the same gesture.

Cho et al. [23] used a median filter to reduce the noise present in an image. This enhanced the accuracy of gesture recognition using skin color detection, and an optical flow gradient operator was then proposed to render the shape of a hand to improve results in hand gesture recognition. A Kalman filter can be used to track hands, and hand recognition is finished using the hidden Markov model (HMM). This method, however, is only applied when there is a simple background.

Wang et al. [24] proposed a new Gaussian model and median filtering model of binary images. Parallel and pipelined hardware architecture were used to reduce the complexity and make hardware implementation easy. However, in most gesture recognition systems, the forearm is considered to be a redundant object that must be removed as the area of the forearm will cause changes in extracted features. This work ignores the removal process of the forearm and thus creates a limitation in the application.

Nunez-Prieto et al. [25] presented a real-time gesture recognition system using a regular phone camera for hand-data acquisition using FPGA implementation as an accelerator. In their work, a CNN was used for classification. However, the most important problem in implementing a neural network in FPGA is that the calculated throughput and memory bandwidth may not match. Due to the insufficient utilization of hardware resources, or the memory bandwidth, existing designs cannot achieve optimal performance.

## 3. The Proposed System

**Figure 1:**
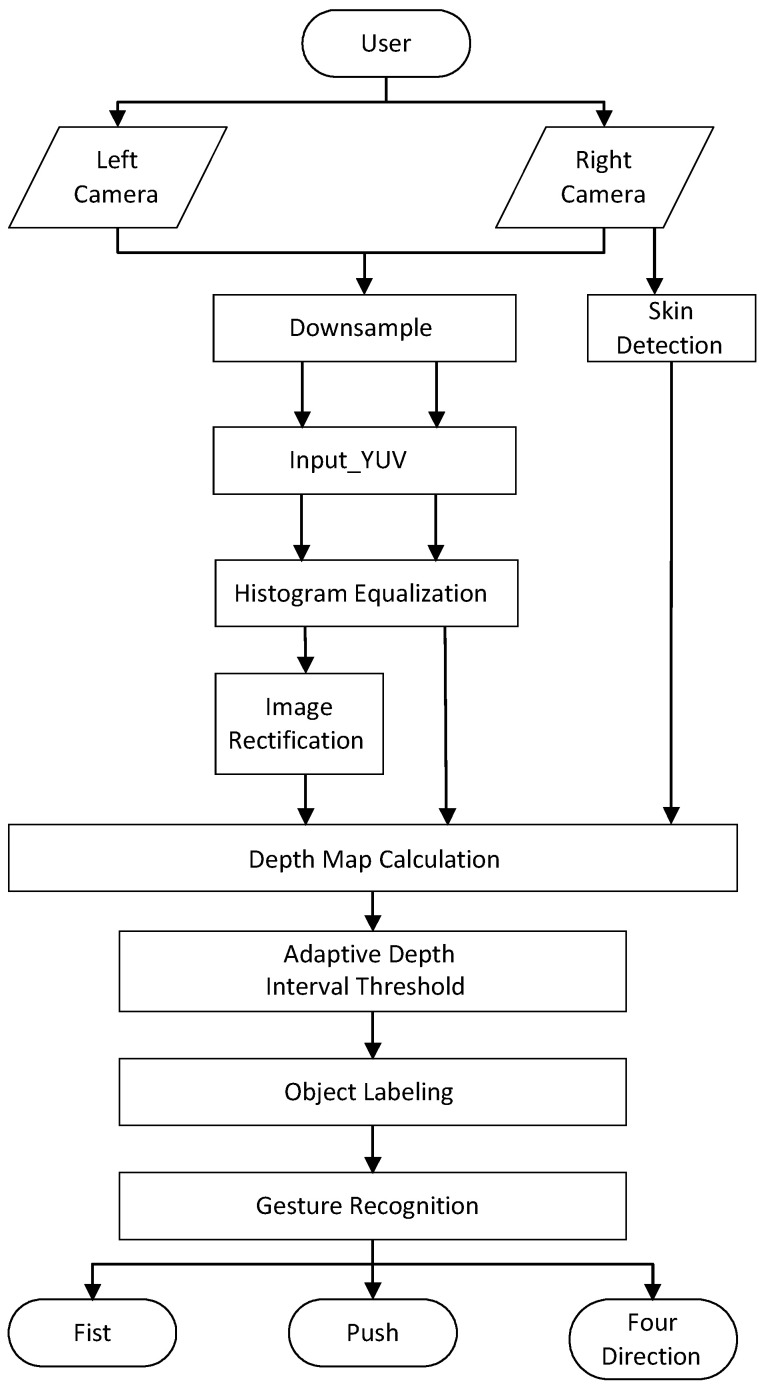
Design flow of the overall system.

**Figure 2:**
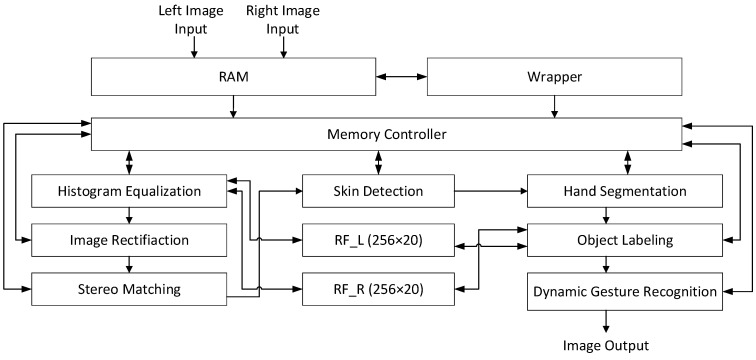
Block diagram of the proposed system.

The hand gesture recognition system proposed by us contains several important modules. The design flow is shown in Figure 1. We captured the two camera inputs as a stereo video for processing. After a series of computation tasks, we could acquire static and dynamic hand gestures. The hardware block diagram is illustrated in Figure 2. The input of the proposed system is YUV data, which were processed by the software when recording the data [26]. Two kinds of memory were used in the system. Off-chip memory (RAM) was used to save the Y value of the left and right image pixels, as well as the U and V values of the right image pixels. A memory wrapper was used to read the images from the RAM. Two 256 × 20 register-file memory modules were used to save some static numbers. The entire design shared these six memory modules to save the different results. More details of the image preprocessing, stereo matching, skin detection, hand segmentation, object labeling, and hand gesture recognition are discussed later. The overall implemented FPGA system will be discussed in Section 4.

### 3.1. Image Preprocessing

The proposed system uses a dual-camera with a stereo matching algorithm to construct a depth map. Many factors affect the accuracy of the depth map, so three preprocessing methods, downsampling, histogram equalization, and image rectification, were applied to enhance the matching efficiency in this system. The image captured by the system’s dual camera was an RGB color image with a size of 1280 × 720. Then we downsampled the image size to 160 × 120 and converted the image information to YUV. Depth information was created and used with grayscale information and did not require color information. Thus, the conversion to the YUV format could further reduce the size of the data file. When a dual-camera device captures an image, it is usually affected by light because the two sensors have different positions, and, as a result, they acquire the different strengths for light, which affect the Y value. To reduce the light effect in the different cameras and to make the pixel value distribution of the two cameras similar, histogram equalization was used to preprocess both images. This process was performed in the first module of the system. The equation of histogram equalization is shown below:

(1) $$\begin{array}{c} s_{k}=T\left(r_{k}\right)=\left(L-1\right)\sum_{j=0}^{k}p_{r}\left(r_{j}\right)=\frac{L-1}{MN}\sum_{j=0}^{k}n_{j},\\ 0\leq r_{k}\leq 1\;\;\;\;k=0,1,\ldots ,L-1, \end{array}$$

**Figure 3:**
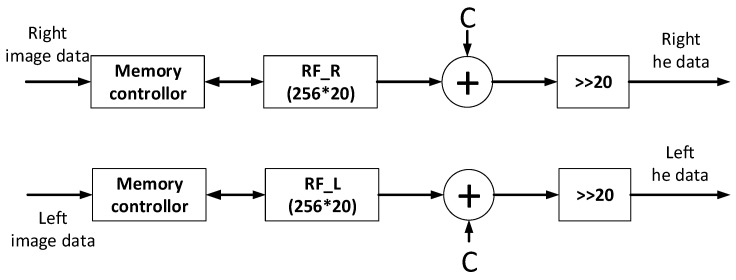
Architecture of histogram equalization.

For the equation, *n_j_* is the histogram for *r_j_* pixel, *L* is 256 as the range of the pixel value is 256, *MN* is the total number of pixels in a frame, and $s_{k}$ is the final gray pixel. Using this formula, all pixels can be assigned a new value. The architecture for histogram equalization is illustrated in Figure 3. The register-file memory was used to save the static numbers. Constant C is used to round the numbers and the shifter is used to replace the divider.

**Figure 4:**
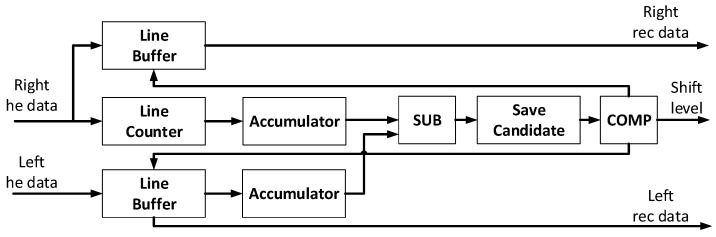
Hardware architecture of image rectification.

The third module is for image rectification. Due to the stereo matching operation, the system needed to fit the left and right images in the epipolar geometry. Image rectification is quite important, especially in dual-camera systems, as it directly affects the accuracy when matching pixels. In this system, we assumed that there is only a horizontal difference between the two cameras. Thus, the system rectifies the left image, i.e., it move up or down to fit the epipolar line in the same horizon. The proposed image rectification algorithm has a low complexity for rectifying the two images. Eight horizontal lines of the right image are selected to match the left image. Each of the horizontal lines of the right image has to match the eleven horizontal lines of the left image. The equations are shown as Equations (2)–(4) [27]. *E* is the total horizontal summation in one row. *E_d_* is the minimum difference in the left and right images for each horizontal line. The system will calculate each minimum difference number for ten lines. Then, the shift level value is calculated through the eight *E_d_* numbers to rectify the left image. The hardware architecture is demonstrated in Figure 4. After calculation of the shift level, a line buffer is used to rectify the image. This module only needs to calculate the shift level in the first frame. For all other frames, the same shift level value is used to rectify the left image.

(2) $$E=\overset{n}{\underset{k=0}{\sum }}I(k),\;\;n=1280$$

(3) $$E_{d}\left(b\right)=min\left\{abs\left|E_{r}\left(b\right)-E_{l}\left(b_{m}\right)\right|\right\},\;\;b=1\sim 8\;\;and\;\;m=-5\sim 5$$

(4) $$Shift\;Level=\frac{\sum_{b=1}^{8}\;min\left\{abs\left|E_{r}\left(b\right)-E_{l}\left(b_{m}\right)\right|\right\}}{8}=\frac{\sum_{b=1}^{8}E_{d}\left(b\right)}{8}$$

### 3.2. Stereo Matching

After preprocessing, the system has two left and right images. Depth information is the key point to determine the area of interest and recognize 3D gestures. In our system, we used the SAD algorithm with a low complexity for stereo matching to construct the depth map [28]. This is because the accuracy of the depth map is not very important; it can tolerate the accuracy of the SAD algorithm in terms of results and can recognize hand gestures. The proposed stereo matching module deals with a 1280 × 720 resolution image with a disparity of 64 with a window size of 5 × 5 pixels for each block. The minimum error value of the SAD algorithm was considered as the disparity of the pixel, and the equation is as (5) [29,30] to illustrate the disparity in the pixels. *I_R_* and *I_L_* are the coordination pixels in the left and right images. Component d means the disparity of the system, so here d is 64. After calculating the disparity, the system will replace the disparity value in map depth pixel in the range of 0 to 255 using (6). The uniform formula is adopted because the non-uniform formula needs dividers to calculate the result. To avoid the usage of the divider, the value of the right-hand side of (6) was utilized through the look-up table while calculating *v*. *Z_near_* is the nearest disparity value, *Z_far_* is the farthest disparity value, *Z_v_* is the current disparity value, *v* is the final depth pixel, and *N* is 256.

(5) $$Z_{v}=\arg min[\overset{160}{\underset{x=1}{\sum }}\overset{120}{\underset{y=1}{\sum }}\left|I_{R}\left(x,y\right)-I_{L}\left(x+d,y\right)\right|]$$

(6) $$\frac{v}{N-1}=\frac{Z_{v}}{Z_{near}-Z_{far}}$$

**Figure 5:**
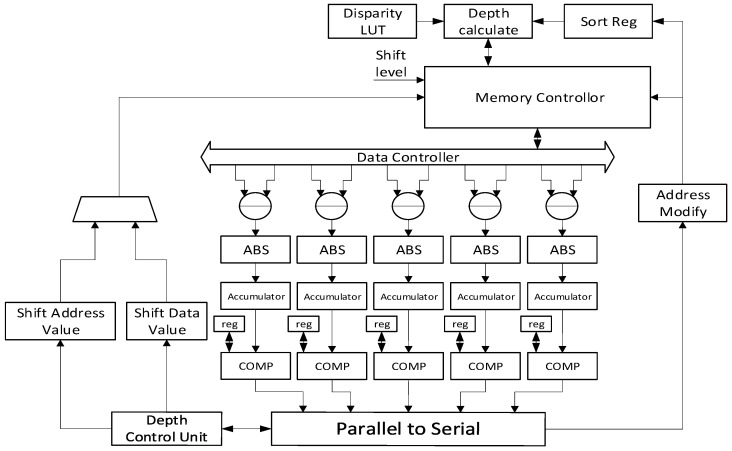
Hardware architecture of stereo matching.

Although the hardware architecture is proposed to speed up the system, the stereo matching module still takes too many clock cycles. Many types of research are attempting to determine how to reduce the overall cycles in a system while calculating SAD error values [31]. The basic concept is that many pixels in the left image are reused when matching with the right image. This allows memory access through the reusing scheme for the left and right images. Based on this scheme, the proposed architecture is applied by shifting the pixel value for the next SAD computation. The proposed system also consists of a parallel architecture with five processing engines. The implementation scheme for the proposed stereo matching is shown in Figure 5. Five engines were used as there are five pixels in one address and this helps to reduce the complexity of the memory data controller and speeds up the overall system. As a result, the overall number of calculation cycles is greatly reduced. The data controller controls the memory and fetches the required pixels to calculate the sum of the difference. The depth control unit controls the stereo matching module, which needs to shift the address to read a new row or shift data values to calculate the disparities of the subsequent five pixels. The disparity LUT is used when transforming a disparity value to a depth value without using a divider.

### 3.3. Skin Detection

After acquiring the depth information, skin detection was performed to remove noise. The system removes non-skin-like colors using the range of the YUV data. In this way, information can be extracted that is related to hands and faces. The range of skin detection values is illustrated by Equation (7) Then, the skin data was passed to the next module. Till now, the system still cannot recognize the hand part because of the face and other skin-like noises.

(7) $$skin\left(x,y\right)=\{\begin{array}{cc} 1,\;\;\;\;if & \{\begin{array}{c} 65<Y<170\\ 85<U<140\\ 85<V<160 \end{array}\\ 0, & \;\;\;else \end{array}$$

### 3.4. Adaptive Dynamic Threshold

After removing the non-skin-like color, the system will find the area of interest, i.e., the hand. Using skin and depth information, hand segmentation can be performed. The proposed hand segmentation checks the statistics of skin color’s depth value. We designed the system to calculate the adaptive dynamic threshold to separate hands and faces through depth information. The horizontal axis is the depth value of the skin color and the vertical axis is the statistic number of each depth value. Through the statistic value, the two higher parts can be seen as the hand and head in the frame; thus, we need to separate them.

**Figure 6:**
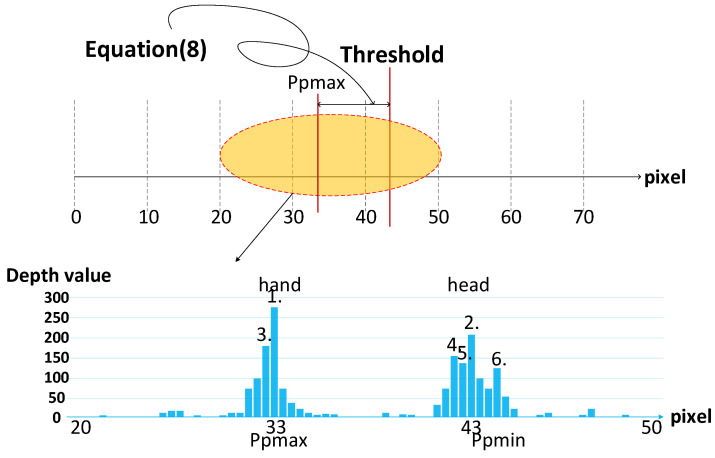
The schematic diagram of adaptive dynamic threshold.

**Figure 7:**
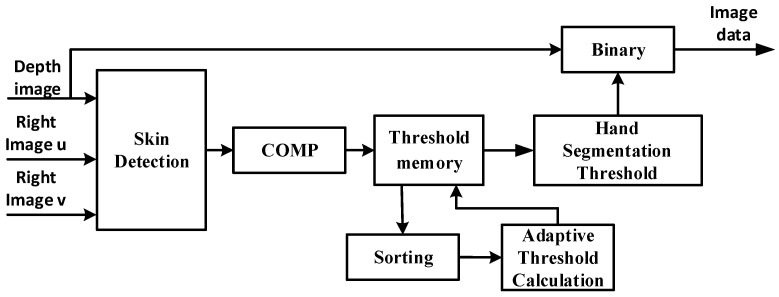
Hardware architecture of hand detection.

The proposed system will pick the six pixels that have the highest probability peaks to calculate the threshold and segment the nearest hand. A schematic diagram of this is shown in Figure 6. We can segment the hand using (8), where *p* is the pixel value after performing the statistics. *d*_1_ and *K* are constants to handle the situation where hand occlusion with face happens or there is only one face or hand in the frame. Using this scheme, the system can still segment the nearest object correctly, regardless of whether there are multiple skin-like color objects or not. The proposed hand detection architecture is illustrated in Figure 7. The statistics are saved in the threshold memory so that the system can find the probability peaks and calculate the threshold.

(8) $$Threshold=\{\begin{array}{l} P_{pmax}-d_{1}\;,\;\;\;\;if\;\left(P_{pmax}-P_{pmin}\right)<K\\ P_{pmax}-\frac{P_{pmax}-P_{pmin}}{2},\;\;\;else \end{array}$$

### 3.5. Object Labeling

The system has acquired the area of interest, i.e., the nearest hand; however, there is still some noise as the foreground object. Object labeling is performed to label foreground objects. In this way, it can help, not only to remove the rest of the noise, but also to calculate the coordinate, length, and width of the hand for the gesture recognition module. For the labeling procedure, the four connected pixels label scheme is applied to perform label assignment. The equation for the labeling scheme is listed in (9). A replacement situation occurs if the X is foreground data and the four connected pixels have more than one label value. Thus, the replacement situation will be saved in the register file memory. After label assignment, some connected labels need to be integrated via label fusion. The proposed system can completely fuse all label values using the following method. First, the memory data are initialized as zero. Then, the replacement situation label value is saved to the original label value address. After that, a table of all the replaced values is generated. The values can be automatically updated by finding the data in the memory through memory addresses. If data are zero, it means that the label value is minimum and does not need to be replaced.

(9) Label(X)=min (label (P), label (Q), label (R), label(S))

**Figure 8:**
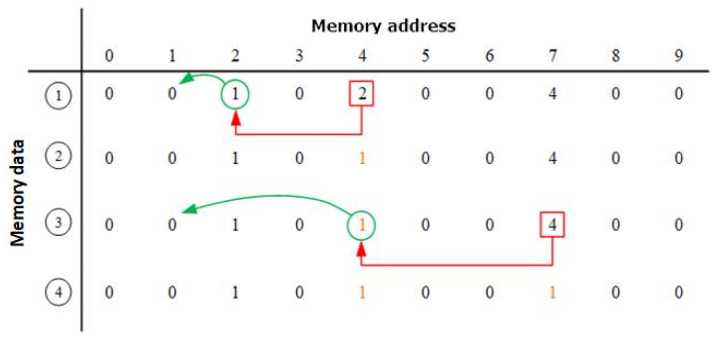
Hardware architecture of hand detection.

**Figure 9:**
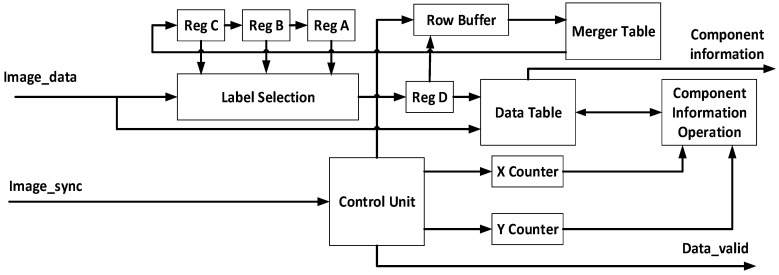
Hardware architecture of object labeling.

Then, two more data values are read from the memory to decide which label value needs to be replaced by a proper label value. A label value similar to the example needs to be replaced, as shown in Figure 8. If one of the two data values is zero, the original value will be replaced with zero; if it is not, the system will update the minimum value of the two values and repeat process again until one of the values is zero. The architecture of object labeling is illustrated in Figure 9. The left part is a label assignment with four connected pixels and the replace situation is saved in a Merger Table.

After finishing the object labeling module, the system has the width, length, and coordination of each object. With this information, the system can remove noise by saving the biggest object to find the hand. Because there is a hand and a small amount of noise at this stage, there is an efficient way to remove noise.

### 3.6. Hand Segmentation

In this module, the system utilizes the information from the hand part and the object labeling module to develop hand gesture recognition information. The proposed system has one static and five dynamic gestures. The static gesture is a fist gesture, which is judged by its contour information through object labeling. Recognition of the fist gesture is elaborated in (10).

(10) $$Fist\;=\;\{\begin{array}{cc} 1 & Object\;length<Object\;width*1.5\\ 0 & Otherwise \end{array}$$

In the proposed system, two dynamic gestures are recognized only with the “fist” situation. The fist gesture can be treated as a trigger of dynamic gestures. When the status is a fist, the system will record the current depth value and calculate the threshold of the push gesture adaptively by adding constant and depth values. If the depth value exceeds the threshold, the system will regard it as a push gesture. The method that updates the initial gesture depth value follows Algorithm 1:
**Algorithm 1**. Pseudocode for the algorithm to determine push gesture thresholdInput: fist, current depth Output: threshold Initialization: count = 0 Begin 1: for i = 1:n do 2:  if(fist = 1 & count = 0) 3:    threshold = current depth +c 4:    count = 1 5:  else if (fist = 1) 6:    threshold = threshold 7:  else count = 0 8: end

With the coordinate information from the hand part, the system can continuously record hand trajectory to recognize “up, down, left, right” gestures using a fist gesture. When the status is not the fist gesture, the system will calculate the weight of the four directions to judge a gesture’s status. The system can also judge the variety of depths to construct a push gesture through the depth information. Whenever the status is the fist status again, the threshold will be updated. The two dynamic gestures can interact with each other to construct “push up”, “push down”, etc. With these signals, products can use our hand gesture recognition to easily control some appliances.

## 4. Experimental Results

In this section, a discussion regarding the two parts of the experiment results is included. The first part includes details about the ASIC design with different specifications, a comparison of the design with other research, and a discussion of the results for each gesture. The second part includes the FPGA implementation and a demonstration of the HCI system.

### 4.1. ASIC Design

**Figure 10:**
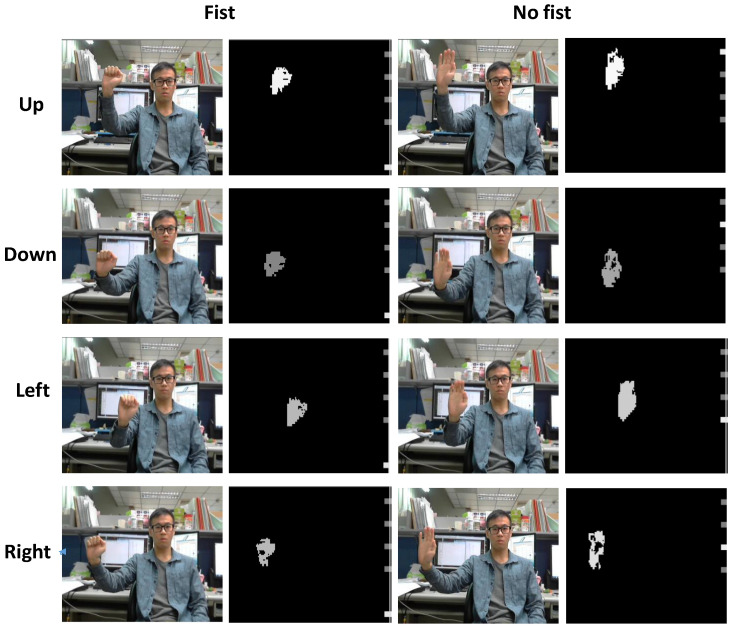
Four directional gesture results.

The video input of the proposed system was recorded using a general dual-camera PC cam. After the data were rearranged and transformed into the YUV format by the software, the data were used as the input pattern. To demonstrate the experiment results and verify all the gestures, several video streams were used to test and verify the system. In the proposed system, the most significant goal was to capture the nearest hand and retrieve information about it. To enhance memory efficiency and reduce power consumption, the two stereo frames were not saved after finishing all the modules. With the trade-off of memory usage, power consumption, and chip efficiency in mind, the resolution of the system was chosen to be 1280 × 720. Figure 10 demonstrates the gesture of “up, down, left, right”, where the left part is the start frame and the right part is the final frame. The gestures from top to bottom are up, down, left, and right, respectively. There are also some images on the right to demonstrate the status. When the system recognizes that the gesture is up, the fist spot will be highlighted, similar to the other gestures. The push and four-direction gestures can interact with each other, so the spot can also help us to verify the gesture of push-up, push-left, and so on.

**Figure 11:**
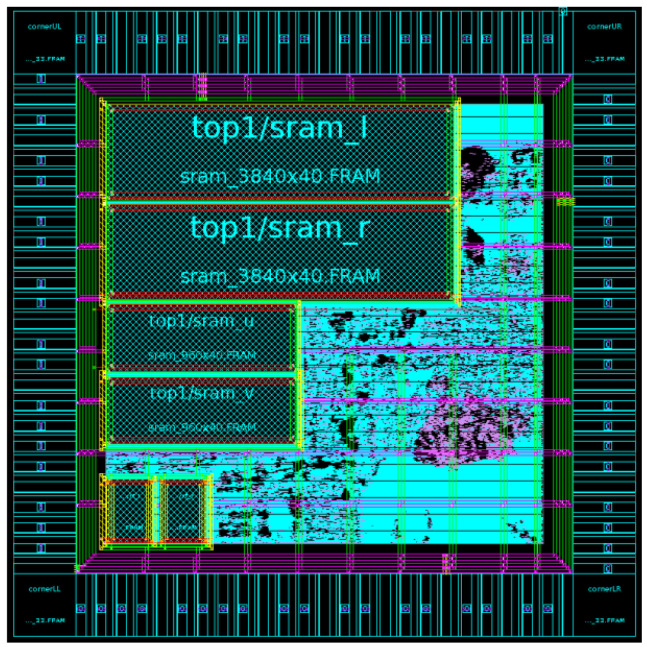
ASIC layout.

A comparison with different studies is listed in Table 1. Our design of the FPGA is synthesized using the same modules from our previous work [10]. Compared with [10], we changed the data flow to reduce the burden of accessing SRAM and optimized the data arrangement. Referring to previous works [20,21], both used the number of spreading fingers for recognition, which is not accurate for gesture recognition. Although dynamic gestures can be recognized (as in [22]), they can only operate in a simpler background. The work in [23] was designed using a CNN for hand recognition. It used a great deal of hardware resources and the FPS was lower than that in other work. Our work can recognize dynamic gestures without being affected by the environment. The Table 1 shows that, when the resolution is 1280 × 720, our FPS was similar to that of other methods. The results showed that it can recognize correct gestures in complex environments while using fewer Slice LUTs than other methods. This means that the proposed architecture can acquire depth information and recognize some gestures on low-cost devices with high efficiency. The specifications of the proposed system are illustrated in Table 2. ASIC design was synthesized for the TSMC 90 nm technology, the operation frequency was 420 MHz to run at 60 frames per second. The gate count and power were 47.3 K and 23.63 mW, respectively. The layout of the chip area was 1.58 × 1.61 mm, with 102 pins, as shown in Figure 11. The total memory requirement was 1.25 kilobytes to temporarily save data during data processing.

**Figure 12:**
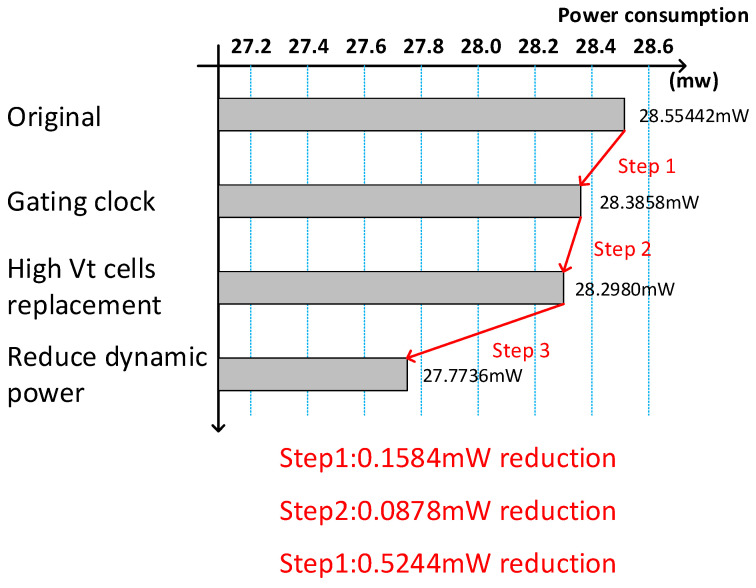
Analysis of power reduction.

We used some techniques to reduce power consumption. Figure 12 illustrates the power reduction, step-by-step, using some techniques. Estimation was evaluated using Synopsys Primepower. As shown in Figure 12, the original power consumption was 28.5442 mW. We applied gated clock technology to reduce the power consumption. The AND logic gate was used to multiply the enable signal and clock. We also used the high Vt replacement to reduce leakage power and dynamic power reduction was also used. Finally, the power consumption of the proposed design was only 27.7736 mW.

### 4.2. FPGA Implementation and Demonstration

**Figure 13:**
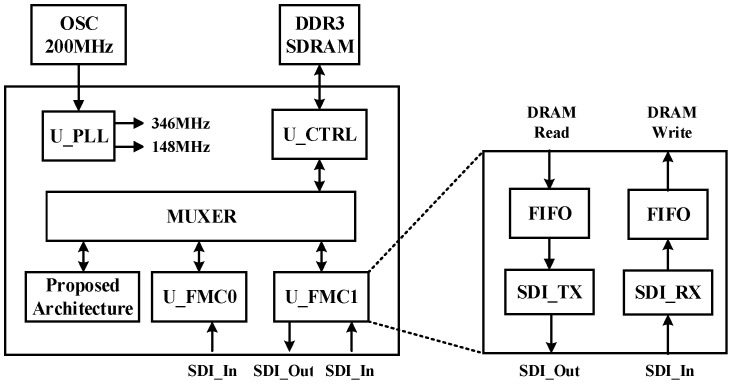
System architecture of FPGA implementation.

To verify the overall system, hardware architecture was implemented in the SMIMS VEXA7-200 development board. The system architecture of FPGA implementation is shown in Figure 13. The development board contained an FPGA with Xilinx Artix-7 and two FMC daughter boards to control the input and output information. Because the image data captured by the cameras were quite large, the system saves the image data in DDR3 memory and outputs the DDR3 data via HDMI to a monitor. Due to the limited bandwidth and operation frequency of DDR3, our design was modified for a resolution of 160 × 120 to meet the constraint limits of DDR3. FIFO was utilized to synchronize the speed of input and the output images and to prevent image data loss. The proposed architecture uses the same multiplexer with two IPs to control the DDR3 controller. Thus, the lowest priority of the proposed system is to prevent input and output image overflow, which would cause a system breakdown.

**Figure 14:**
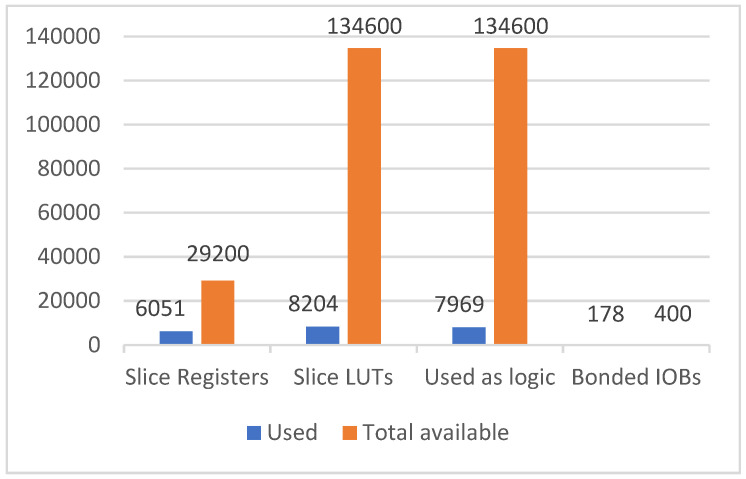
The resource usage of FPGA.

The resource usage of the FPGA is illustrated in Figure 14. The average accuracy of the proposed system is 83.98%, and is shown in Table 3. To test the system gesture recognition rate, there were 10 different users for measuring the system gesture recognition rate and each gesture was performed 100 times.

**Figure 15:**
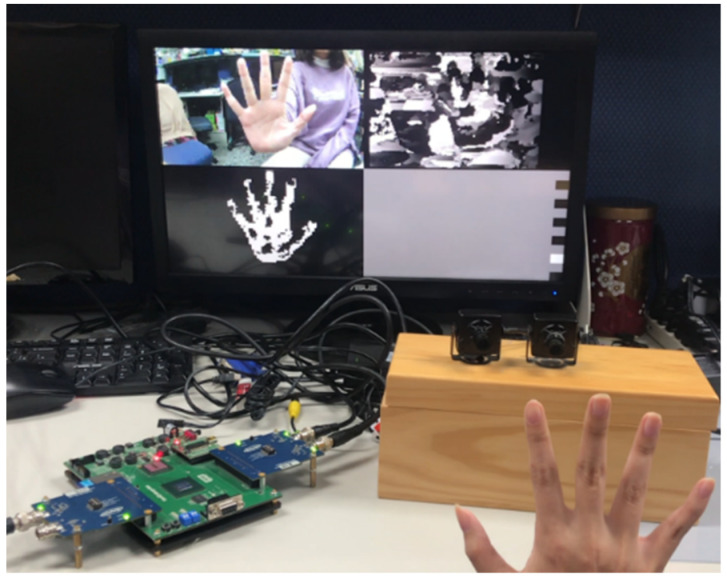
The real demonstration system.

A real demonstration system for the overall proposed work was constructed, as shown in Figure 15. It was implemented in the SIMIS development board. A dual-camera device was used to capture the video. After the entire process, the output result is shown on a monitor to achieve the goal of the HCI. Note that we did not have a clean background to make hand segmentation easier. We ran the program in an environment with a complex background.

## 5. Conclusions

In this paper, a VLSI hardware architecture system with a dual-camera is proposed. We construct the depth map with a stereo matching algorithm and recognize hand gestures to meet the goal of 3D hand gesture recognition. The preprocessing module is selected to increase the accuracy of stereo matching. The SAD algorithm is applied to match the pixels and construct the depth map. We calculate the adaptive threshold to separate hands and faces using the depth information, and also remove most of the noise. Then object labeling processing was performed on the information to remove the rest of the noise. Several gestures in the video have been simulated in this system. We have created a complete VLSI design with each architecture module. This whole system is also implemented in FPGA with the SMIMS development board to demonstrate the HCI system and to verify the overall architecture. The dynamic gestures can be combined with each other so they can be applied with to some home appliances and control them easily. The average accuracy of all gesture recognition is 83.98%. Based on the proposed system, several hand gestures can be recognized, even in a complicated environment.

## Figures and Tables

**Table 1:** The comparison of each researcher.

	Raj [20]	Cho [21]	Wang [22]	Núñez-Prieto [23]	This Work
Camera	Single-camera	Single-camera	Single-camera	Single-camera	Dual camera
Platform	Virtex-4 ML402	ALTERA Cyclone II EP2C70	Virtex-5 XC5VLX50T	xcku060	SMIMS VEXA7-200
Remarks	Skin-detection Centroid calculation Finger count determination	Skin-color-detector Optical flow operator Kalman filter	Skin-detection Median filter Fingertip detection	CNN ZynqNet	SAD Skin-detection Object-Labeling Trajectory-detection
Image size	--	640 × 480	640 × 480	--	1280 × 720
Frequency	--	125 MHz	N/A	200 MHz	420 MHz
Frame rate	--	75FPS	60FPS	23.5FPS	60FPS (180FPS@640*480)
Throughput (pixel per second)	--	230.4 M	180.3 M	15.4 M	553 M
Slice Register	14,053	7251	6421	71,000	6051 (2%)
Slice LUTs	39,540	16,806	12,633	293,000	8204 (6%)
Bonded IOBs	463	447	22	--	178 (44%)
Gesture classes	5 static	2 static 4 dynamic	5 static	29 static	1 static, 5 dynamic (2Dx4 + 3Dx1)

**Table 2:** Specification of the proposed system.

Item	Specification
**Technology**	TSMC 90 nm
**Voltage**	1.0 V / 3.3 V (Core / IO)
**Operation frequency**	420 MHz
**Chip area**	1.580 mm × 1.610 mm
**Core area**	1.020 mm × 1.048 mm
**Gate count**	47.3 K
**Memory requirement**	1.25 KBytes
**Power consumption**	27.7736 mW
**Total pins**	102 pins

**Table 3:** The recognition rate of the proposed system.

Gesture	Samples	Hit	Miss	Average
**Fist**	1000	912	88	91.2%
**Push**	1000	848	152	84.8%
**Four direction**	1000	813	187	81.3%
**Push + Four direction**	1000	786	214	78.6%
**Total**	4000	3359	641	83.98%

## Data Availability

Not applicable.
